# A multi-dataset data-collection strategy produces better diffraction data

**DOI:** 10.1107/S0108767311037469

**Published:** 2011-10-18

**Authors:** Zhi-Jie Liu, Lirong Chen, Dong Wu, Wei Ding, Hua Zhang, Weihong Zhou, Zheng-Qing Fu, Bi-Cheng Wang

**Affiliations:** a National Laboratory of Biomacromolecules, Institute of Biophysics, Chinese Academy of Sciences, Beijing 100101, People’s Republic of China; b Department of Biochemistry and Molecular Biology, University of Georgia, Athens, GA 30602, USA

**Keywords:** multi-dataset data-collection strategy, readiness test

## Abstract

A multi-dataset (MDS) data-collection strategy is proposed and analyzed for macromolecular crystal diffraction data acquisition. The theoretical analysis indicated that the MDS strategy can reduce the standard deviation (background noise) of diffraction data compared with the commonly used single-dataset strategy for a fixed X-ray dose. In order to validate the hypothesis experimentally, a data-quality evaluation process, termed a readiness test of the X-ray data-collection system, was developed. The anomalous signals of sulfur atoms in zinc-free insulin crystals were used as the probe to differentiate the quality of data collected using different data-collection strategies. The data-collection results using home-laboratory-based rotating-anode X-ray and synchrotron X-ray systems indicate that the diffraction data collected with the MDS strategy contain more accurate anomalous signals from sulfur atoms than the data collected with a regular data-collection strategy. In addition, the MDS strategy offered more advantages with respect to radiation-damage-sensitive crystals and better usage of rotating-anode as well as synchrotron X-rays.

## Abstract 2

Theoretical analysis and experimental validation prove that a multi-dataset data-collection strategy produces better diffraction data. The readiness test is a simple and sensitive method for X-ray data-collection system evaluation and a benchmark.

## 1. Introduction

The X-ray diffraction data of crystals contain the critical three-dimensional structural information of the crystallized molecules; they are the only direct experimental source for subsequent elucidation of spatial structures of the crystallized molecules. The X-ray diffraction data collection of single crystals refers to the process of measuring diffracted intensities and their standard deviations (noise) from single crystals. The quality of the diffraction data determines the accuracy of the final model. For macromolecular crystallography, there are many factors that compromise the data quality. The factors can be categorized into three groups. (i) Crystal: the diffraction quality is based on the internal degree of order of the molecules and the mosaicity of the crystal, and the cryo-freezing status such as the selection of cryo solution, loop and cryo treatment. (ii) Instrumentation: the X-ray beam quality (monochromaticity, intensity/position stability, divergence *etc.*), goniometry (mechanical accuracy of the goniometer system and shutter synchronization) and the quality of the detectors [dark current correction, balance of different mosaic chips, sensitivity, dynamic range, detective quantum efficiency (DQE) *etc.*]. (iii) Data-collection strategy: the wavelength, attenuation, detector-to-crystal distance, exposure time, start angle, scan range and oscillation angle. Therefore, for a given crystal and X-ray data-collection system, the key to obtaining the highest possible quality of diffraction data lies in the data-collection strategy (Cianci *et al.*, 2008 ▶; Sarma & Karplus, 2006 ▶).

When compared to crystals of small molecules, macromolecular crystals diffract X-rays poorly and usually tend to have a much shorter lifetime in the X-ray beam. In other words, a macromolecular crystal can only withstand a certain amount of X-ray dose before it is destroyed as a result of radiation damage. Therefore, obtaining accurate and complete diffraction data sets of macromolecular crystals within their lifetime is very important (González, 2003 ▶; Leal, 2011 ▶; Yang *et al.*, 2003 ▶).

In this study, a multi-dataset (MDS) data-collection strategy is proposed. The theoretical analysis indicates that the MDS data-collection strategy at a fixed X-ray dose produces better-quality data. In order to validate the hypothesis experimentally, a data-quality evaluation process, termed a readiness test of the X-ray data-collection system, was developed. Zinc-free insulin crystals were used as the standard testing crystals and the anomalous signals of sulfur atoms in insulin crystals were used as an indicator to differentiate the quality of data collected using the different data-collection strategies.

## 2. A look at the theory

In a traditional data-collection experiment, the crystal is exposed *x* s per frame and a total of *y*° is scanned. The proposed MDS strategy involves *x*/*N* s per frame of exposure (*N* is a positive integer) while scanning a total of *y*°, where the scanning is repeated *N* times. In terms of X-ray dosage, both strategies put the same amount of X-ray photons into the crystal, but the MDS strategy produces better-quality data. Let’s take a look at the theory.

In the 1960s, the single counting diffractometers were developed for X-ray analysis of crystals of small molecules. The standard deviation values of the reflections were calculated by 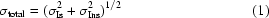

            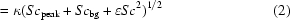
where σ_total_ is the total standard deviation of the measured reflection spot, σ_Is_ is the standard deviation of the counting statistics and σ_Ins_ is the standard deviation of the instrument error. *Sc*_peak_ and *Sc*_bg_ are photon counts for the reflection peak region and the background region, respectively. *Sc* is the sum of photon scan counts, ∊ is experimental (ignorance) factor, generally 0.02 < ∊ < 0.10. When area detectors were developed for macromolecular crystal data collections in the 1980s, the standard deviation value of individual reflections from the two-dimensional area detectors was also modeled by the two types of errors expressed in equation (1). For example, 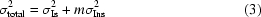

            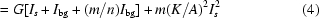
where *G* is the gain of the detector, *m* and *n* are the number of pixels in the reflection peak region and background region of the measurement box, respectively, *I*_*s*_ and *I*_bg_ are the summation intensity of peak and background, respectively, *K* is a proportionality constant, and *A* is a factor which is related to the half-width of a reflection spot (Leslie, 2001 ▶).

It is obvious that the value of σ_total_ increases rapidly with an increase in *I*_*s*_. Now, if we reduce the exposure time by a factor of *N*, such that 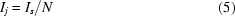
where *I*_*j*_ is the summation of peak intensity during 1/*N* exposure time, then
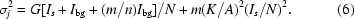
We compensate for the weaker data by repeating the data collection *N* times. Adding the intensities of all the equivalent reflections together, we get 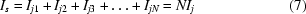

            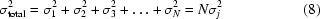

            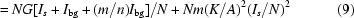

            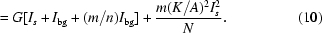
According to equation (7), in theory, it is possible to recover intensities for reflections using the MDS strategy as with the regular data-collection strategy. Remarkably, the MDS strategy for data collection reduces errors [second term in equation (10)] by a factor of *N* when compared to data collected using the regular method [equation (4)]. Therefore, for a fixed X-ray dose, because of the reduction in standard deviation, collecting multiple data sets with the MDS strategy can produce more accurate data than collecting a single data set using the regular data-collection method.

## 3. Data-quality evaluation

The difference between the data collected with the regular and MDS strategies can turn out to be marginal and therefore a sensitive method is required to measure the subtle difference and assess the impact of this difference on the structure solution. We decided to use sulfur’s anomalous signal in zinc-free insulin crystals as a probe to assess the data quality of diffraction data collected using both strategies. Sulfur’s anomalous signal is comparatively weak if the diffraction data are collected using the usual X-ray wavelength (0.97–2.0 Å), but this shortcoming has not stopped researchers from using sulfur’s anomalous signal as a phasing probe. It has been explored experimentally by Hendrickson & Teeter (1981 ▶) and theoretically by Wang (1985 ▶). More successful cases were reported in the 1990s (Dauter *et al.*, 1999 ▶; Liu, 2000 ▶). Therefore, sulfur atoms’ weak anomalous signal can serve as a sensitive probe to distinguish the subtle difference in the diffraction data collected with different strategies. The efficiencies of the two data-collection strategies can be evaluated by measuring and comparing the strengths of the anomalous signal recorded in the diffraction data. The rationale for choosing insulin crystals is as follows: (i) a Zn-free insulin crystal has high symmetry (*I*2_1_3 space group) and is suitable for collecting data with both strategies without introducing too much radiation damage to the crystal; (ii) it is easy to obtain an insulin sample and grow crystals, and the diffraction resolution (around 2.0 Å) of an insulin crystal is suitable for the evaluation of data quality; (iii) there are three disulfide bonds per insulin molecule and the anomalous signal from those three disulfide bonds is a perfect probe for the evaluation of data quality. Three parameters were proposed to evaluate the quality of the data collected using the different strategies:

(1) Relative peak height (RPH): RPH is the ratio of the average peak height of three disulfide bonds (the top three highest peaks) and the average peak height of the last three (seventh, eighth and ninth) in the first nine highest peaks in the anomalous difference Fourier map calculated at 50.0–2.5 Å resolution using anomalous data and rigid-body-refined model phases calculated by the program *FFT* in the *CCP4* suite (Collaborative Computational Project, Number 4, 1994 ▶). The idea here is to compare the anomalous peak densities of the three ‘specific’ disulfide bonds (top three) in relation to the ‘representative’ noise peaks in the map. It is expected that a higher RPH value means stronger anomalous signals from three disulfide bonds were recorded and thus a set of better-quality data was collected.

The fourth, fifth and sixth highest peaks were not selected in the calculation because of the consideration that they may be more affected by experimental conditions. For example, any metal ions from either the insulin sample, buffer or crystallization solutions may contribute to the higher level of background anomalous signals. Therefore, peaks 4, 5 and 6 are more likely to be affected than are peaks 7, 8 and 9; in other words, the seventh, eighth and ninth peaks are more eligible to ‘represent’ the noise level in the map.

(2) Map correlation coefficient (Map cc): Map cc is the map correlation coefficient between the model-phased 2*f*_o_ − *f*_c_ electron-density map and the S-SAD-phased experimental map calculated at the same resolution range (50.0–2.5 Å). It is used to measure the deviations between the experimentally S-SAD-phased map and the theoretically calculated ideal map. It is an indirect indication of the data quality collected using different strategies. The model-phased map was calculated using the Fourier synthesis method with equation (11): 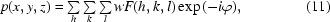
where *p* is the electron-density function, *w* is the figure of merit (FOM) calculated from the rigid-body refinement process, *F* is the difference of the two times’ measured amplitude in the diffraction data minus the calculated diffraction factor (2*f*_o_ − *f*_c_), ϕ represents the phases calculated from the refined model. The S-SAD experimentally phased map was calculated using the same equation (11) and the same amplitude *F*, but the FOM and phases were calculated using sulfur atoms’ anomalous scattering signals in each data set (Wang, 1985 ▶). The sulfur atoms’ coordinates were obtained from the rigid-body-refined models. The Map cc is the correlation coefficient between two maps, calculated using *Overlapmap* in the *CCP4* suite (Collaborative Computational Project, Number 4, 1994 ▶). It is defined by equation (12) 
            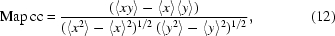
where *x* represents the density values from one map and *y* represents the values from the other map, 〈〉 represents the mean value of the quantities inside the parentheses.

(3) Ratio of map correlation coefficient (Rcc): Rcc is defined as the ratio of Map cc calculated for data collected using the MDS strategy to the Map cc of data collected using the regular strategy and is expressed as 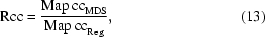
where Map cc_MDS_ and Map cc_reg_ are the map correlation coefficients of data collected with the MDS and regular strategies, respectively, of the same crystal. It is designed to compare the effectiveness of MDS and regular data collection. A larger value of Rcc indicates a bigger difference between the two data-collection strategies.

## 4. Experimental validations

### 4.1. Crystallization and data collection

The bovine pancreas insulin sample was purchased from Sigma–Aldrich (catalog No. I5500). In order to obtain Zn-free insulin, the insulin sample was dissolved in buffer (50 m*M* NaHPO_4_, 0.02 m*M* Na_3_EDTA, pH 11.0) to a final concentration of 15 mg ml^−1^, then dialyzed against the buffer (0.018 *M* Na_2_HPO_4_, pH 10.5 with 0.001 *M* EDTA pH 9.0) overnight; the buffer was changed three times every 4 h. The crystallization experiment was carried out using the hanging drop vapor diffusion method: 2 µl hanging drops containing 1 µl protein mixed with 1 µl mother liquor were equilibrated over 300 µl reservoir solution and incubated at 289 K. Crystals were grown in 15% PEG 4000, 100 m*M* Bis-Tris, pH 8.0 and 100 m*M* NaCl. The insulin crystals with size of around 0.2 × 0.2 × 0.2 mm were soaked in mother liquor containing 30% glycerol for 5 s before flash freezing in liquid nitrogen for subsequent diffraction testing and data collection. The anomalous diffraction data were collected using both home-laboratory copper rotating-anode and synchrotron X-ray sources with a wavelength of 2.00 Å. The rotating-anode diffraction data were collected using a Saturn 944+ CCD detector with MicroMax-007 X-ray generator. The synchrotron data were collected using 2.00 Å wavelength X-rays at the 22-ID beamline (SER-CAT), Advanced Photon Source (APS), Argonne National Laboratory.

Each crystal was used for data collection twice – first with a regular exposure time followed by one third of the exposure time but with the data collection repeated three times at the same scan range. The overall X-ray dosages for both regular- and MDS-exposed data were the same. Three insulin crystals with a similar size and diffraction quality were tested for each data-collection strategy. In order to demonstrate that the MDS data-collection approach can truly produce better-quality data than the regular approach, even with some less favorable con­ditions, the regular-exposure data were collected first. The rationale behind the approach is as follows. The theoretical analysis indicated that the data collected with the MDS strategy are of better quality than the data collected with the regular strategy. If the data with the regular collection strategy were collected with fresh crystals, which was then followed by data collection with the MDS strategy, the data quality produced with the MDS strategy should be compromised by the radiation damage incurred during the regular data collection. If, even in such a less favorable case, the MDS strategy still produces data with superior quality compared with those of the regular strategy, then the theoretical prediction is proved and the artifact of radiation damage during different measurement is avoided. If the order of data collection for the regular and MDS strategies is reversed, the artifact of radiation damage cannot be eliminated and the conclusion that the MDS strategy is better may not be reached.

### 4.2. Structure determination and calculations

Data collected with rotating-anode X-rays were indexed and scaled using *HKL2000* (Otwinowski & Minor, 1997 ▶). The data-collection and data-processing results are listed in Table 1 ▶(*a*). Data collected with synchrotron X-rays were indexed and integrated using d*TREK (Pflugrath, 1999 ▶), and scaled using *3DSCALE* (Fu *et al.*, 2004 ▶). The data-collection and data-processing results are listed in Table 1 ▶(*b*). The structure was solved by a difference Fourier method using *REFMAC* (Murshudov *et al.*, 1997 ▶) in the *CCP4* suite (Collaborative Computational Project, Number 4, 1994 ▶) with porcine insulin (PDB code 9ins) as an initial model (Gursky, 1992 ▶). In order to minimize the model bias on the calculations, only ten cycles of rigid-body refinement were carried out for each data set using *REFMAC* (Murshudov *et al.*, 1997 ▶) at the 50.0–2.5 Å resolution range. There are two possibilities when the cubic insulin crystal is indexed and only one of them complies with the porcine insulin crystal structure deposited in the PDB as 9ins. The other index can be converted with the matrix [−*K*, *H*, *L*].

## 5. Results

### 5.1. The relative peak height – RPH

Six crystals were selected for data collection on two different detectors with two types of X-ray sources. Crystals 1, 2 and 3 were collected on a Rigaku Saturn944+ CCD detector while crystals 4, 5 and 6 were collected on a Mar 225 CCD detector at the 22-ID synchrotron beamline of SER-CAT at APS, Argonne National Laboratory, using 2.00 Å wavelength X-rays. All six crystals diffracted X-rays beyond 2.0 Å resolution. Since the strength of anomalous signals from sulfur atoms decreases with the increase in diffraction resolution, all the calculations were planned to be performed within the 50.0–2.5 Å resolution range and therefore the data-collection parameters were chosen to ensure the high-resolution ends of the data were at least 2.30 Å (0.2 Å resolution margin was set during the data-scaling process). The parameters are detector size, crystal-to-detector distance, exposure time and X-ray wavelengths. The scan ranges for crystals 1, 2 and 3 were 50° for each data-collection path. The exposure time for the first data set (regular-exposure data set) of crystals 1, 2 and 3 was 45 s while the subsequent three data sets (MDS-exposure data set) were collected three times at the same scan range with a 15 s exposure time for each data set. The crystals were not translated between the regular and MDS data collections for the sake of minimizing the influence of diffraction variations at different locations of the crystals. The same data-collection strategy was applied to crystals 4, 5 and 6. The regular exposure time, collected at the synchrotron for crystals 4, 5 and 6, was 9 s while the exposure time for the MDS data set was 3 s. The scan ranges were 90°. For each crystal, the reflections for regular-exposure data were indexed, integrated and scaled into one data set while the reflections for the three MDS-exposure data sets were merged and scaled into one data set. The relative peak height for each data-collection strategy was calculated and is listed in Table 2 ▶. As expected, the redundancy and *I*/σ_*I*_ value of the MDS-exposed (MDS strategy) data are significantly higher than those of the regular-exposed data for all crystals. The relative peak height of MDS-exposed data is higher than that of the regular-exposed data.

### 5.2. The map correlation coefficient – Map cc

The subsequent calculations for the map correlation coefficient revealed that the MDS data yield a better map compared with the regular-exposure data in terms of the agreement between the model-phased map and the S-SAD-phased map. This result indicates that the sulfur atoms’ anomalous signal was more accurately recorded in the MDS data than in the regular-exposed data. The map correlation coefficient values Map cc and Rcc for both types of data-collection strategies of the six crystals are listed in Table 2 ▶.

**Figure 1:**
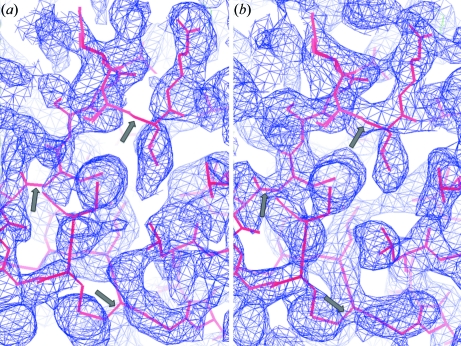
The superposition of the rigid-body-refined insulin molecule model and the S-SAD-phased experimental 2*f*_o_ − *f*_c_ electron-density map at 50.0–2.5 Å resolution contoured at 1.0σ. (*a*) The map was calculated using the regular-exposed data of crystal 1. The arrow signs in the figure indicate the missing density at the main-chain area. (*b*) The map was calculated using the MDS-exposed data of crystal 1.

The regular and MDS diffraction data from crystal 1 were selected to calculate the S-SAD-phased 2*f*_o_ − *f*_c_ electron-density map at 50.0–2.5 Å resolution as shown in Fig. 1 ▶. The map quality of the MDS data is clearly better than that of the regular-exposed data, which agrees with the Map cc values.

## 6. Discussion

In this study, a multi-dataset data-collection strategy is proposed and analyzed for macromolecular crystal diffraction data acquisition. The theoretical analysis indicated that the MDS strategy can reduce the standard deviation of diffraction data when compared to the single-dataset strategy for a fixed X-ray dose. The benefits of the MDS strategy are the result of the multiple measurements of the same set of diffraction spots *versus* fewer measurements in a regular data-collection strategy. For example, in a regular single-dataset data-collection experiment, each frame is exposed for *x* s, while in an MDS data-collection experiment each frame is exposed *x*/*N* s, but the whole scan range is repeated *N* times. The crystal receives the same amount of X-ray dose in both data-collection strategies. But from equation (10), it is obvious that the second term of standard deviation is reduced by *N* times in the MDS strategy; thus the MDS strategy produces more accurate data than collecting a single data set using the regular data-collection method.

In order to experimentally verify the theoretical predictions of the MDS strategy, a sensitive and simple method is developed to determine the difference between the diffraction data collected using both strategies. The calculations from the diffraction data of six insulin crystals collected using two different data-collection systems showed that the diffraction data collected with the MDS strategy are obviously better than those collected by the regular single-path strategy in terms of the three parameters used in the data-quality evaluations as shown in Table 2 ▶. The comparison of map quality between S-SAD-phased 2*f*_o_ − *f*_c_ electron-density maps at 50.0–2.5 Å resolution calculated from the data of crystal 1 showed the MDS data contain more accurate anomalous signal from sulfur atoms than the data collected with the regular data-collection strategy as shown in Fig. 1 ▶.

The diffraction data quality is determined by two objective factors, the crystal quality and data-collection instrumentation, and one subjective factor, the data-collection strategy. Based on the theoretical analysis and experimental verification, for a macromolecular crystal diffraction data-collection experiment, the MDS data-collection strategy produces better-quality data. In addition, the MDS strategy has other advantages. (i) If the crystal is sensitive to radiation damage, or in the case of micro-focused synchrotron beam data-collection experiments where the radiation damage is more problematic, the MDS strategy offers a better option to obtain more complete data owing to its shorter exposure time for each scan, in addition to better data quality. One can decide on how many scans to be included during the scaling process and eliminate the images which may have suffered too much radiation damage. (ii) Since the MDS strategy uses multiple scans *versus* a single scan in a regular data-collection experiment, the anomalous signal of phasing probes present in the crystal becomes stronger as the number of scans increases, assuming the crystal is reasonably resistant to radiation damage. This offers the enhanced opportunity for carrying out signal-based data collection (Rose *et al.*, 2007 ▶), in which the data collection, data processing and monitoring of the anomalous signal are calculated ‘on-the-fly’ during the data-collection process. The objective of signal-based data collection is to obtain a pre-set anomalous signal from phasing probes, including the use of additional crystals automatically mounted by a robot if necessary, and data collection will not stop until there is enough of the required anomalous signal for a successful phasing of the structure. (iii) With the new advances in X-ray detection technology, more sensitive and low-noise detectors such as pixel array detectors are being adopted in macromolecular crystal data collection. Taking advantage of these kinds of detectors, researchers may use much shorter exposure time to obtain similar signal-to-noise ratios when compared with traditional CCD detectors. Thus these kinds of detectors coupled with the MDS strategy can help researchers obtain a much higher quality of diffraction data. (iv) The MDS data-collection strategy can be employed for in-house data collection using a rotating-anode X-ray source because the relatively weaker X-ray beam intensity is more suitable for the multiple data-collection experiments. The application may include S-SAD using Cu or Cr rotating-anode X-rays, Se or intrinsic metal SAD experiments using either Cu/Cr rotating-anode or synchrotron X-rays as well. One good example is the crystal structure determination of human ferrochelatase where Fe-SAD was used. The anomalous signal from the 2Fe–2S cluster was not strong enough to solve the structure until the data redundancy reached 70-fold (Wu *et al.*, 2001 ▶). (v) The readiness test of the X-ray data-collection system developed in the study is sensitive and simple enough for serving the purpose of differentiating the quality of data collected by different strategies. But the readiness test has broader usage in the following area: (*a*) it can serve as a standard X-ray data-collection system evaluation tool. It can be used routinely as a benchmark to test the status of the performance of the whole X-ray data-collection system. (*b*) It can be used as an optimization tool for choosing optimal experimental parameters for sulfur phasing such as wavelength, attenuation, crystal-to-detector distance, exposure time *etc*.

An important consideration while performing MDS data-collection experiments is that the selection of minimum exposure time should ensure that the photon counts are within the detector’s linear response range.

In conclusion, the theoretical analysis and experimental verifications support the contention that the MDS data-collection strategy offers a better chance to acquire higher diffraction data quality. The readiness test of the X-ray data-collection system is a sensitive and simple tool for X-ray system evaluation and optimization. We hope more researchers may try this new type of data collection strategy and improve it further.

## Figures and Tables

**(a):** Crystals 1, 2 and 3. X-ray source: Rigaku MicroMax-007. X-ray optics: VariMax HR; detector: Rigaku Saturn 944+; wavelength: 1.54 Å; space group: *I*2_1_3.

	Crystal 1		Crystal 2		Crystal 3	
Cell dimensions: *a* = *b* = *c* (Å)	77.96		77.59		78.42	
Exposure (s)	45.0	15.0	45.0	15.0	45.0	15.0
Scan range (°)	50.0	3 × 50.0	50.0	3 × 50.0	50.0	3 × 50.0
Resolution (Å)†	50.00–2.00 (2.07–2.00)	50.00–2.00 (2.07–2.00)	50.00–1.95 (2.02–1.95)	50.00–2.10 (2.18–2.10)	50.00–1.95 (2.02–1.95)	50.00–2.10 (2.18–2.10)
*R*_sym_ (%)	5.3 (22.7)	5.5 (44.5)	4.8 (33.7)	6.9 (38.8)	3.9 (23.5)	5.8 (48.8)
*I*/σ_*I*_	47.84 (6.4)	66.21 (6.18)	39.60 (4.71)	53.16 (10.06)	42.07 (5.58)	51.52 (5.17)
Completeness (%)	99.6 (99.8)	99.8 (100.0)	93.5 (61.1)	98.4 (90.9)	99.8 (100.0)	98.4 (90.9)
Redundancy	5.3	16.0	5.5	16.9	5.2	15.5

**(b):** Crystals 4, 5 and 6. X-ray source: SER-CAT 22-ID; X-ray optics: monochromator; detector: Mar 225 CCD; wavelength: 2.0 Å; space group: *I*2_1_3.

	Crystal 4		Crystal 5		Crystal 6	
Cell dimensions: *a* = *b* = *c* (Å)	77.84		78.58		77.76	
Exposure (s)	9.0	3.0	9.0	3.0	9.0	3.0
Scan range (°)	90.0	3 × 90.0	90.0	3 × 90.0	90.0	3 × 90.0
Resolution (Å)‡	50.00–2.30 (2.38–2.30)	50.00–2.30 (2.38–2.30)	50.00–2.30 (2.38–2.30)	50.00–2.30 (2.38–2.30)	50.00–2.30 (2.38–2.30)	50.00–2.30 (2.38–2.30)
*R*_sym_ (%)	5.2 (8.9)	6.5 (12.1)	5.3 (7.7)	5.8 (10.0)	5.1 (11.1)	6.7 (17.6)
*I*/σ_*I*_	62.3 (45.3)	89.4 (58.0)	69.8 (55.1)	106.5 (69.8)	58.2 (37.5)	105.7 (96.0)
Completeness (%)	99.24 (99.14)	99.38 (99.19)	99.14 (99.05)	99.36 (99.19)	99.46 (99.30)	99.41 (99.29)
Redundancy	10.3	30.8	10.2	30.3	10.3	30.4

† Numbers in parentheses are statistics for the highest-resolution shell.

‡ Data were processed with ‘d*TREK’ then scaled by ‘*3DSCALE*’ software.

**Table 2:** Anomalous signal calculation RPH: relative peak height is the ratio of the average peak height of peaks 1, 2 and 3 divided by the average peak height of peaks 7, 8 and 9 in the anomalous difference map calculated at 50.0–2.5 Å resolution. Map CC: map correlation coefficient between the S-SAD-phased map and the model-phased map at 50.0–2.5 Å resolution. Rcc: ratio of Map CC between the MDS data and the regular-exposed data of the same crystal.

	Crystal 1		Crystal 2		Crystal 3		Crystal 4		Crystal 5		Crystal 6	
	Regular	MDS	Regular	MDS	Regular	MDS	Regular	MDS	Regular	MDS	Regular	MDS
Resolution (Å)	50.0–2.5		50.0–2.5		50.0–2.5		50.0–2.5		50.0–2.5		50.0–2.5	
RPH	1.66	2.46	2.96	3.19	2.92	3.19	2.43	2.64	2.42	2.54	2.33	2.55
Map cc	0.37	0.53	0.58	0.61	0.52	0.66	0.767	0.804	0.726	0.757	0.787	0.839
Rcc	1.43		1.05		1.27		1.05		1.05		1.27	
